# Evaluating prevalence and risk factors of building-related symptoms among office workers: Seasonal characteristics of symptoms and psychosocial and physical environmental factors

**DOI:** 10.1186/s12199-017-0645-4

**Published:** 2017-04-12

**Authors:** Kenichi Azuma, Koichi Ikeda, Naoki Kagi, U Yanagi, Haruki Osawa

**Affiliations:** 1 Department of Environmental Medicine and Behavioral Science, Kindai University Faculty of Medicine, 377-2 Ohnohigashi, Osakasayama, Osaka 589-8511 Japan; 2 Department of Architecture, College of Science and Technology, Nihon University, 8-14 Kanda-Surugadai 1-chome, Chiyoda-ku, Tokyo, 101-8308 Japan; 3 Department of Mechanical and Environmental Informatics, Graduate School of Information Science and Engineering, Tokyo Institute of Technology, 2-12-1 Ookayama, Meguro-ku, Tokyo, 152-8550 Japan; 4 Department of Architecture, School of Architecture, Kogakuin University, 1-24-2 Nishi-Shinjuku, Shinjuku-ku, Tokyo, 163-8677 Japan; 5 Department of Environmental Health, National Institute of Public Health, 2-3-6 Minami, Wako, Saitama 351-0197 Japan

**Keywords:** Building-related symptoms, Cross-sectional study, Humidity control, Indoor air quality, Sick Building Syndrome, Occupational stress

## Abstract

**Background:**

Psychosocial and environmental factors at the workplace play a significant role in building-related symptoms (BRSs). Environmental factors change during summer cooling and winter heating using air-conditioning systems. Thus, significant risk factors in each season need to be clarified.

**Methods:**

A nationwide cross-sectional study was conducted during summer in Japan and seasonal differences between summer and winter were evaluated. Self-administered questionnaires were distributed to 489 offices. Possible risk factors for BRSs associated with the work environment, indoor air quality, and job stressors were examined by multiple regression analyses.

**Results:**

Among people having at least one BRS, the prevalence of BRSs in summer (27.8%) was slightly higher than that in winter (24.9%). High prevalence was observed for eye and nasal symptoms related to dryness and general symptoms related to psychological distress in both seasons. Analyses revealed that dryness of air was an important and significant risk factor associated with BRSs, and job stressors were significantly associated with general symptoms in both seasons. Conversely, humidity was a significant risk factor of general symptoms in summer (odds ratio, 1.20; 95% confidence interval, 1.02–1.43). Carpeting, recently painted walls, and unpleasant chemical odors in summer and noise, dust and dirt, and unpleasant odors such as body or food odors in both seasons were significant risk factors for BRSs.

**Conclusions:**

Improvements in the physical environmental qualities in an office throughout the year are important along with the reduction in psychological distress related to work.

**Electronic supplementary material:**

The online version of this article (doi:10.1186/s12199-017-0645-4) contains supplementary material, which is available to authorized users.

## Background

Nonspecific building-related symptoms (BRSs), commonly called sick building syndrome, have emerged as an environmental and occupational health issue [1]. BRSs relate to situations in which building occupants suffer from respiratory (stuffy and irritated nose, rhinitis, cough, sore throat, and shortness of breath), ocular, skin, and general (fatigue, headache, and fever) symptoms, and these symptoms are relieved when the person is away from the building [2, 3]. Personal factors, including gender [4, 5] and personality traits [6]; environmental factors, such as poorly maintained ventilation systems and poor humidification systems [7, 8]; indoor environmental quality; the work environment [4, 9–19]; and occupational stress [5, 14, 20–23] have been found to be associated with BRSs.

In Japan, the Building Sanitation Management Standards, which specify management standards for the maintenance of indoor air quality, water supply and drainage, cleaning, and pest control were established in 1970. However, the proportion of buildings that do not conform to the standards of relative humidity, room temperature, and carbon dioxide has increased in the last decade in Japan [24]. In addition, development of eye irritation associated with visual display unit work (i.e., computer-related job) in workplaces with low humidity [25, 26] and development of upper airway inflammation associated with exposure to particles emitted from photocopiers or laser printers used in the workplace [27] have been suggested.

We firstly conducted a nationwide cross-sectional questionnaire survey on possible risk factors associated with BRSs in office workers working in office buildings during winter in Japan [28]. We also estimated the prevalence of BRSs among office workers in Japan. In the survey, people having at least one BRS accounted for 25% of the respondents. BRSs were associated with multiple factors, including work environment (carpeting and crowded workspaces), indoor air quality (perception of coldness, perception of air dryness, unpleasant odors, and reported dustiness on the floor), and occupational stress (amount of work and interpersonal conflicts). However, several factors change during summer cooling and winter heating using air-conditioning and heating systems, respectively. In particular, indoor air concentrations of specific volatile organic compounds, including strong irritants of the upper respiratory tract and skin, may increase in summer [29–31]. The relationships between these factors are complicated. Moreover, the proportion of buildings that did not conform to the Building Sanitation Management Standard for relative humidity in their offices has been found to increase in winter [32]. Therefore, significant risk factors in winter (cold season) and summer (hot season) must be clarified. Following the survey conducted in winter, we conducted a nationwide cross-sectional questionnaire survey in summer to evaluate the prevalence and risk factors of BRSs and evaluated the seasonal characteristics.

## Methods

### Study design and population

The same study design and the subjects as described in our previous study [28] were used to compare seasonal characteristics. Of 2882 companies belonging to the 47 local prefectural associations of the Japan Building Maintenance Association, 489 companies [four to 50 companies (average 10.4 company) per prefectural association] were recommended by the prefectural association on the basis of the number of companies in a given association. This ensured an even representation across all prefectures in Japan. Each company office was selected from a different building. The two questionnaires, one for the office managers and the other for office workers, working in the office, which was used in our previous study [28] were prepared. The study design was cross-sectional. The participation of office workers was anonymous and voluntary; participants expressed consent to participate through their completion of the questionnaire.

Office workers, such as managers, planning and administrative staff, communication engineers, and designers, who regularly spend long hours in the office during the daytime were selected by the office manager as participants within the office; however, those who were engaged in cleaning the building or measuring indoor air quality were excluded. The overall sizes of the offices in a building included in the study were small because of the small number of office workers who were usually located on one floor alone. These criteria limited the selection of participants. We therefore relied on the office managers to select all office workers when 10 or less office workers in their office met the selection criteria. Otherwise, managers arbitrarily selected 15 office workers at a maximum that met the criteria. Each office manager received a questionnaire to complete, and distributed an additional 15 questionnaires to be completed by their office workers. Surveys were conducted from August to October 2012 (summer) after the previously conducted winter surveys (January to March 2012).

### Questionnaires

As described in detail previously [28], in the questionnaire designed for office managers, the office managers were asked to provide basic information about their offices and the buildings in which they were located, including the total floor area of the building and its year of construction. The questionnaire designed for office workers comprised the United States Environmental Protection Agency (USEPA) Questionnaire for indoor environmental quality survey [33], the Indoor Air Questionnaire (MM-40) [34], and the Brief Job Stress Questionnaire [35, 36]. We added items regarding installed office equipment, mold odor in workplace, and pet ownership at home.

The office workers were asked about their gender, age, job category, smoking status, contact lens use, specific symptoms, frequency of symptoms, association of symptoms with the building, and their perception of the work environment, indoor air quality, and occupational stress. With regard to the work environment, we asked participants the following: the number of people working in the room in which workstation of respondent is located, the condition of their workstation (carpeting, lighting, experience of reflection or glare in the field of vision, table comfort, and chair comfort), computer use, frequency odorous chemical use, any change within five meters of their workstation within the last 3 months (new carpeting, painted walls, furniture, partitions, wall covering, or water damage), the equipment within two meters of the workstation (laser printer, bubble jet printer, copier, exterior window, and door), and indoor workplace installations (fragrance, air freshener, and repellent). In the questionnaire about specific symptoms, we presented a page-long table of symptoms. For each symptom, respondents chose how often the symptom had occurred while working in the building in the last 4 weeks and whether or not the symptom had improved after they left work. In the questionnaire about the perception of indoor air quality, questions were also presented in a page-long table. For each question, respondents chose how often the workplace environmental conditions, such as heat or cold, humidity or dryness, noise, and odors, had been experienced while working in the building during the last 4 weeks.

The job stressor scale comprised 17 general items related to job stress that were rated on a four-point Likert scale ranging from yes (1) to no (4). Responses related to amount of work, mental workload, physical overload, interpersonal conflict, environmental stress, job control, skill utilization, job suitability, and work satisfaction were converted to job stressors using a score translation table. Environmental stress was not included in the following analyses because it is related to other environmental variables such as work environment and perception of indoor air quality.

### BRS groups

As described in detail in our previous study [28], analyses included weekly BRSs, defined as specific symptoms that a person experienced in the building at least 1 day per week in the last 4 weeks and that improved when the person was away from the building. The BRSs include eye irritation, general symptoms, upper respiratory symptoms, lower respiratory symptoms, and skin symptoms. The eye irritation included dry or irritated eyes and tired eyes. The general symptoms included headache, unusual tiredness, tension, difficulty concentrating or remembering things, dizziness, feeling depressed, and nausea. The upper respiratory symptoms included sore or dry throat, sinus congestion, cough, and sneezing. The lower respiratory symptoms included wheezing, chest tightness, and shortness of breath. The skin symptoms included dryness, itching, and irritation of the skin. Thus, 19 symptoms were investigated in total. The symptom group consisted of persons who reported at least one BRS.

### Statistical analyses

While examining the possible risk factors associated with weekly BRSs, the same procedure as in our previous study [28] was used in this study. We examined correlations (Spearman’s test) between the variables for multicollinearity by creating a correlation matrix and then scanning for highly correlated variables (≥0.7). To prevent multicollinearity, highly correlated variables were not included in the multiple logistic regression model [37, 38]. Univariate associations between BRSs and potential risk factors were examined, and factors with *p* < 0.2 were selected for multiple logistic regression analysis.

As described in detail in our previous study [28], personal factors and job stressors were tested using multiple logistic regression analyses to determine potential risk factors associated with BRSs (Model 1). The associations between BRSs and the work environment, adjusting for personal and job stressors, were analyzed (Model 2). The associations between BRSs and the workplace conditions in the last 4 weeks, adjusting for personal factors and job stressors, were analyzed (Model 3). Finally, the selected potential risk factors were included in a stepwise logistic regression analysis (forward selection with Wald statistics) to identify independent risk factors for BRSs (Model 4). The *p*-values for entry and removal of variables in the stepwise logistic regression model were 0.05 and 0.1, respectively. Goodness of fit was measured with the chi-square test and Hosmer and Lemeshow tests [39].

For examining seasonal differences (between winter and summer) in the prevalence of BRSs, the odds ratio (OR) based on the odds of summer versus those of winter was calculated. Data of our previous study [28] were used for this analysis. Adjusted OR, adjusted for potential confounders of personal factors, which are *p* < 0.2 in univariate analyses between winter and summer, was calculated using the Mantel-Haenszel method.

We used *p* < 0.05 to indicate statistical significance. ORs and 95% confidence intervals (CIs) were determined for univariate and multivariate associations, and the seasonal comparisons of prevalence. All data analyses were performed using IBM SPSS version 22 for Windows (IBM Corp, Armonk, New York).

## Results

### Participants

Of the original 489 offices, 309 provided office worker responses (response rate, 63.2%) and 307 provided responses from office managers (response rate, 62.8%). Two offices provided responses from their office workers but not from the managers. Questionnaire responses were obtained from 3024 office workers, with an average of 9.8 (SD = 4.1) participant office workers per office.

Characteristics of the participants are shown in Table 1. The mean age was 44.2 years (range, 19–78 years), 38.0% were women, and most participants had planning or administrative jobs. The mean duration of employment was 7.6 years. The proportions of buildings by year of construction were 9.0%, 22.0%, 18.0%, 36.9%, and 14.1% in 1950–1969, 1970–1979, 1980–1989, 1990–1999, and ≥2000, respectively. Approximately 51% of the buildings surveyed in this study were built after 1990. The proportions of building in terms of its total floor area were 47.3%, 20.5%, 6.7%, 11.7%, 9.4%, and 4.4% for <1000 m^2^, 1000 to <3000 m^2^, 3000 to <5000 m^2^, 5000 to <10,000 m^2^, 10,000 to <50,000 m^2^, and ≥50,000 m^2^, respectively.

**Table 1:** Participant characteristics (*N* = 3024)

Characteristic	Mean ± SD or n/N (%) ^*a*^
Gender	
Male	1859/2998 (62.0)
Female	1139/2998 (38.0)
Age group	
10–19	6/2990 (0.2)
20–29	352/2990 (11.8)
30–39	807/2990 (27.0)
40–49	794/2990 (26.6)
50–59	645/2990 (21.6)
≥ 60	386/2990 (12.9)
Job categories	
Managerial	656/2947 (22.3)
Professional	139/2947 (4.7)
Technical	415/2947 (14.1)
Sales	363/2947 (12.3)
Planning/administrative	1223/2947 (41.5)
Secretarial/clerical	6/2947 (0.2)
Other	145/2947 (4.9)
Smoking status	
Never	1286/3006 (42.8)
Former	714/3006 (23.8)
Current/sometime	99/3006 (3.3)
Current/everyday	907/3006 (30.2)
Mean year working in the building (*n* = 2997)	7.6 ± 7.1

^*a*^ Data for characteristics were missing for some participants (no response)

### Prevalence of BRSs

The prevalence of 19 health symptoms related to work environments are shown in Table 2. Weekly BRS is defined as symptoms experienced at least 1 day per week in the last 4 weeks that improved when one was away from the building. Monthly BRS shows symptoms that are experienced at least 1 day in the last 4 weeks that improved when one was away from the building.

**Table 2:** Prevalence of health symptoms related to work environments (*N* = 3024)

Symptoms	Weekly^*a*^ n/N (%) ^*b*^	Monthly^*c*^ n/N (%) ^*b*^
Tension, irritability, or nervousness	327/2825 (11.6)	245/2825 (8.7)
Tired or strained eyes	286/2800 (10.2)	171/2800 (6.1)
Dry, itching, or irritated eyes	222/2952 (7.5)	121/2952 (4.1)
Feeling depressed	195/2857 (6.8)	179/2857 (6.3)
Unusual tiredness, fatigue, or drowsiness	186/2911 (6.4)	143/2911 (4.9)
Sore or dry throat	108/2919 (3.7)	108/2919 (3.7)
Headache	78/2938 (2.7)	168/2938 (5.7)
Difficulty remembering things or concentration	62/2837 (2.2)	104/2837 (3.7)
Sneezing	52/2828 (1.8)	102/2828 (3.6)
Cough	36/2902 (1.2)	84/2902 (2.9)
Dry or flushed facial skin	36/2974 (1.2)	37/2974 (1.2)
Stuffy or runny nose, or sinus congestion	34/2893 (1.2)	63/2893 (2.2)
Hands dry, Itching, red skin	33/2963 (1.1)	26/2963 (0.9)
Dizziness or lightheadedness	32/2928 (1.1)	65/2928 (2.2)
Nausea or upset stomach	32/2941 (1.1)	65/2941 (2.2)
Scaling/itching scalp or ears	23/2961 (0.8)	26/2961 (0.9)
Shortness of breath	15/2978 (0.5)	32/2978 (1.1)
Chest tightness	15/2995 (0.5)	49/2995 (1.6)
Wheezing	7/3003 (0.2)	17/3003 (0.6)
Total^*d*^	721/2597 (27.8)	1330/2666 (42.4)

^*a*^ A participant experienced the symptom at least 1 day per week in the last 4 weeks that improved when the participant was away from the building

^*b*^ Data for characteristics were missing for some participants (no response)

^*c*^ A participant experienced the symptom at least 1 day in last 4 weeks that improved when the participant was away from the building

^*d*^ At least one of 19 symptoms reported by participants

The prevalence of weekly BRSs in terms of eye irritation, general symptoms, upper respiratory symptoms, lower respiratory symptoms, and skin symptoms was 14.1%, 18.3%, 6.7%, 0.9%, and 2.2%, respectively. The prevalence of those symptoms (weekly), irrespective of whether the symptoms improved when away from work, was 29.3%, 31.8%, 22.2%, 3.6%, and 8.0%, respectively. Thus, the proportions of symptoms related to the work environment were 48.1%, 57.5%, 30.0%, 24.8%, and 27.0%, respectively. In the BRSs, the prevalence of lower respiratory symptoms was very low; so, this symptom group was excluded from subsequent modeling.

### Risk factors associated with BRSs

We examined the correlations among 53 variables (7 personal factors, 22 work environment factors, 16 indoor air quality factors, and 8 job stressors). No highly correlated variables (correlation ≥ 0.7) existed. Univariate associations between BRSs and all personal and other variables, and the numbers of cases by variable factors for weekly BRSs are presented in the Additional file 1 (Table S1 and Table S2, respectively). The subsequent results of multiple logistic regression analysis models for the association with weekly BRSs are shown in Table 3 (Model 1), Table 4 (Model 2), Table 5 (Model 3), and Table 6 (Model 4), and the results of the risk factors associated with weekly BRSs are described below.

**Table 3:** The association of weekly building-related symptoms with personal factors and job stressors (Model 1)

Variable factors	Eye irritation OR (95% CI)	General symptoms OR (95% CI)	Upper respiratory OR (95% CI)	Skin symptoms OR (95% CI)
	*N* = 2590	*N* = 2505	*N* = 2486	*N* = 2687
Personal				
Gender (female)	2.17 (1.53–3.09)^**^	2.19 (1.57–3.05)^**^	2.33 (1.42–3.83)^**^	5.05 (2.10–12.14)^**^
Age				
10–19	1.45 (0.15–14.02)	1.14 (0.12–10.68)	2.98 (0.29–30.11)	–
20–29	2.06 (1.15–3.67)^*^	2.34 (1.37–3.99)^**^	1.69 (0.77–3.69)	0.74 (0.25–2.16)
30–39	1.65 (0.96–2.84)	1.74 (1.07–2.85)^*^	1.40 (0.67–2.92)	0.68 (0.27–1.75)
40–49	1.26 (0.73–2.17)	1.12 (0.68–1.84)	0.87 (0.41–1.86)	0.46 (0.17–1.24)
50–59	1.76 (1.02–3.03) ^*^	1.18 (0.71–1.96)	1.13 (0.53–2.42)	0.37 (0.13–1.06)
≥ 60	Ref.	Ref.	Ref.	Ref.
*p* for trend	0.048	<0.001	0.149	0.425
Job categories				
Managerial	Ref.	Ref.	Ref.	Ref.
Professional	0.61 (0.27–1.36)	0.77 (0.39–1.52)	0.83 (0.23–3.04)	0.60 (0.07–5.37)
Technical	0.49 (0.28–0.86)^*^	0.76 (0.48–1.19)	1.35 (0.61–2.97)	0.56 (0.11–2.96)
Sales	0.67 (0.41–1.10)	0.90 (0.58–1.39)	1.06 (0.46–2.46)	1.06 (0.25–4.54)
Planning/administrative	1.17 (0.78–1.75)	1.25 (0.84–1.85)	2.39 (1.20–4.76)^*^	1.72 (0.56–5.29)
Secretarial/clerical	2.16 (0.33–14.34)	0.78 (0.08–7.65)	8.85 (1.29–60.90) ^*^	–
Other	0.50 (0.20–1.24)	1.02 (0.51–2.03)	1.96 (0.70–5.46)	2.19 (0.55–8.64)
Smoking				
Never	Ref.	Ref.	Ref.	Ref.
Former	0.91 (0.65–1.27)	0.99 (0.72–1.35)	1.22 (0.77–1.93)	1.77 (0.87–3.63)
Current/sometime	0.99 (0.48–2.06)	0.75 (0.35–1.58)	1.08 (0.40–2.95)	1.09 (0.14–8.43)
Current/everyday	0.93 (0.68–1.27)	0.89 (0.66–1.20)	0.95 (0.60–1.50)	1.54 (0.74–3.18)
Contact lens use	1.49 (1.14–1.95)^**^	1.20 (0.92–1.57)	1.46 (1.00–2.12)^*^	1.18 (0.63–2.19)^*^
Pet ownership (Cat)			1.79 (1.07–2.99)^*^	3.16 (1.59–6.28)^**^
Job stressors				
Amount of work^*a*^	1.24 (1.08–1.42)^**^	1.27 (1.11–1.44)^**^	1.28 (1.09–1.49)^**^	
Mental workload^*a*^	1.19 (1.01–1.39)^*^	1.19 (1.03–1.39)^*^		
Physical overload^*b*^	0.77 (0.65–0.91)^**^	0.93 (0.79–1.08)	1.06 (0.84–1.33)	
Interpersonal conflict^*a*^	1.16 (1.01–1.33)^*^	1.69 (1.47–1.94)^**^	1.25 (1.04–1.51)^*^	1.72 (1.27–2.32)^**^
Job control^*a*^	0.91 (0.80–1.04)	0.77 (0.68–0.87)^**^	0.94 (0.78–1.12)	0.91 (0.68–1.21)
Skill utilization^*c*^	0.84 (0.71–0.98)^*^	0.95 (0.81–1.11)	0.87 (0.70–1.07)	1.06 (0.74–1.51)
Job suitability^*d*^	0.96 (0.82–1.12)	1.12 (0.96–1.30)	1.02 (0.82–1.26)	
Work satisfaction^*d*^	0.97 (0.83–1.14)	0.68 (0.58–0.79)^**^	0.84 (0.67–1.05)	1.02 (0.77–1.36)

Values are expressed as adjusted odds ratios (95% CI) for participants with complete data. Variables with *p* < 0.2 in univariate analyses are included in a multivariate logistic regression analysis. Ref. = referent. Significant at ^*^ *p* < 0.05, ^**^ *p* < 0.01. Text in parentheses reflects case groups

^*a*^ Five levels of response are 1) less/low, 2) somewhat less/low, 3) medium, 4) somewhat more/high, and 5) more/high

^*b*^ Four levels of response are 1) somewhat less/low, 2) medium, 3) somewhat more/high, and 4) more/high

^*c*^ Four levels of response are 1) less/low, 2) somewhat less/low, 3) medium, and 4) somewhat more/high

^*d*^ Four levels of response are 1) less/low, 2) somewhat less/low, 3) medium, and 4) more/high

**Table 4:** Associations between weekly building-related symptoms and the work environment (Model 2)

Variable factors	Eye irritation OR (95% CI)	General symptoms OR (95% CI)	Upper respiratory OR (95% CI)	Skin symptoms OR (95% CI)
	*N* = 2332	*N* = 2275	*N* = 2262	*N* = 2438
No. of people in office^*a*^	1.16 (1.00–1.36)	1.13 (0.97–1.31)	1.11 (0.89–1.38)	1.15 (0.81–1.64)
Work station				
Floor carpet (with)	1.53 (1.13–2.06)^**^	1.56 (1.17–2.08) ^**^	1.77 (1.13–2.78) ^*^	3.07 (1.30–7.27) ^*^
Lighting^*b*^	0.77 (0.58–1.01)	0.89 (0.68–1.17)	0.64 (0.45–0.93)^*^	0.62 (0.35–1.09)
Reflection or glare in vision^*c*^	1.42 (1.23–1.66)^**^	1.48 (1.27–1.72)^**^	1.49 (1.22–1.82) ^**^	1.61 (1.21–2.16) ^**^
Table comfort^*d*^	1.03 (0.82–1.31)	1.07 (0.85–1.35)	0.88 (0.63–1.22)	1.31 (0.78–2.18)
Chair comfort^*d*^	1.48 (1.19–1.86)^**^	1.58 (1.27–1.96)^**^	2.06 (1.53–2.79)^**^	1.18 (0.72–1.95)
Work with computer	3.53 (1.03–12.18) ^*^	1.41 (0.66–3.03)		
Use of odorous chemicals^*e*^	1.14 (1.05–1.23)^**^	1.10 (1.02–1.19)^*^	1.06 (0.94–1.19)	0.98 (0.81–1.19)
Change in workplace^*f*^				
New carpeting	0.99 (0.36–2.75)		0.52 (0.11–2.40)	
Painted wall			5.96 (1.16–30.64)^*^	
New furniture	0.88 (0.48–1.63)		1.06 (0.44–2.56)	
New wall covering	1.19 (0.43–3.28)		1.19 (0.22–6.47)	
Water damage				1.32 (0.40–4.35)
Equipment/installation				
Laser printer^*g*^	1.11 (0.83–1.50)	0.96 (0.72–1.29)		
Bubble jet printer^*g*^	1.05 (0.79–1.40)	1.02 (0.77–1.36)		1.71 (0.92–3.18)
Copier^*g*^	1.25 (0.92–1.70)	1.33 (0.99–1.78)	1.20 (0.81–1.77)	1.64 (0.88–3.07)
Exterior window^*g*^			0.79 (0.54–1.16)	
Door^*g*^	0.71 (0.52–0.97)			
Fragrance^*h*^				1.59 (0.59–4.26)
Air freshener^*h*^		1.04 (0.71–1.44)	1.15 (0.67–1.98)	
Repellent^*h*^			1.15 (0.66–2.03)	0.63 (0.23–1.72)

Values are expressed as adjusted odds ratios (95% CI) for participants with complete data. Values are adjusted for personal factors and job stressors with *p* < 0.2 in univariate analyses using multivariate regression analysis. Ref. = referent. Significant at ^*^ *p* < 0.05, ^**^ *p* < 0.01. Text in parentheses reflects case groups

^*a*^ Number of people working in the room in which workstation of respondent is located. Six levels of response are 1) 1 person, 2) 2–3 persons, 3) 4–7 persons, 4) 8–20 persons, 5) 21–50 persons, and 6) ≥ 51 persons

^*b*^ Five levels of response are 1) much too dim, 2) a little too dim, 3) Just right, 4) a little too bright, and 5) much too bright

^*c*^ Five levels of response are 1) rarely, 2) occasionally, 3) sometimes, 4) fairly often, and 5) very often

^*d*^ Four levels of response are 1) very comfortable, 2) reasonably comfortable, 3) somewhat uncomfortable, and 4) very uncomfortable

^*e*^ Five levels of response are 1) never, 2) less than 3 times/week, 3) 3–4 times a week, 4) about once a week, and 5) several times a day; with cleanser, glue, correction fluid, or other odorous chemicals

^*f*^ Change taken place within five meters of workstation in last 3 months

^*g*^ Within two meters of workstation

^*h*^ In workplace indoors

**Table 5:** Association between weekly building-related symptoms and workplace conditions in the last 4 weeks (Model 3)

Variable factors^*a*^	Eye irritation OR (95% CI)	General symptoms OR (95% CI)	Upper respiratory OR (95% CI)	Skin symptoms OR (95% CI)
	*N* = 2587	*N* = 2502	*N* = 2483	*N* = 2684
Too much air movement	0.78 (0.55–1.09)	0.95 (0.69–1.32)	1.00 (0.66–1.53)	0.61 (0.31–1.23)
Too little air movement	1.00 (0.87–1.15)	1.31 (1.14–1.50)^**^	1.23 (1.03–1.49)^*^	1.13 (0.84–1.54)
Too hot	1.09 (0.94–1.26)	1.15 (1.00–1.33)	1.26 (1.03–1.54)^*^	1.13 (0.80–1.60)
Varying room temperature	1.16 (0.98–1.36)	1.00 (0.85–1.18)	1.03 (0.82–1.29)	1.11 (0.77–1.60)
Too cold	0.97 (0.80–1.18)	1.38 (1.13–1.68)^**^	1.12 (0.87–1.44)	0.91 (0.61–1.35)
Air too humid	1.21 (1.02–1.45) ^*^	1.12 (0.94–1.34)	0.89 (0.69–1.13)	0.98 (0.67–1.43)
Air too dry	1.49 (1.25–1.77)^**^	1.23 (1.02–1.47)^*^	1.78 (1.44–2.19)^**^	2.56 (1.83–3.57)^**^
Static electricity	1.38 (1.01–1.90)^*^	1.08 (0.76–1.53)	0.68 (0.43–1.05)	1.10 (0.63–1.90)
Noise	1.23 (0.99–1.52)	1.50 (1.22–1.84)^**^	1.34 (1.03–1.76) ^*^	1.22 (0.81–1.86)
Airflow from air conditioner	1.11 (0.96–1.28)	1.11 (0.96–1.28)	1.13 (0.93–1.36)	0.89 (0.64–1.25)
Odors from air conditioner	1.07 (0.83–1.39)	1.01 (0.77–1.32)	0.94 (0.67–1.31)	1.25 (0.75–2.09)
Mold odor	0.90 (0.66–1.24)	0.84 (0.61–1.16)	1.09 (0.75–1.58)	0.64 (0.35–1.17)
Dust and dirt	1.08 (0.88–1.32)	1.10 (0.90–1.34)	1.22 (0.96–1.55)	0.82 (0.55–1.22)
Tobacco smoke odor	1.22 (1.03–1.43)^*^	1.23 (1.04–1.45) ^*^	1.19 (0.97–1.46)	1.23 (0.88–1.70)
Unpleasant chemical odor	1.03 (0.69–1.56)	0.87 (0.57–1.32)	1.56 (1.01–2.42) ^*^	2.52 (1.44–4.42)^**^
Unpleasant other odor^*b*^	1.19 (1.00–1.42)	1.37 (1.15–1.64)^**^	1.20 (0.96–1.50)	1.55 (1.10–2.19)^*^

Values are expressed as adjusted odds ratios (95% CI) for participants with complete data. Values are adjusted for personal factors and job stressors with *p* < 0.2 in univariate analyses using multivariate regression analysis. Ref. = referent. Significant at ^*^ *p* < 0.05, ^**^ *p* < 0.01. Text in parentheses reflects case groups

^*a*^ Four levels of response are 1) never, 2) 1–3 days, 3) 1–3 days per week, and 4) every or almost every workday

^*b*^ For example, body odor, food odor, or perfume

**Table 6:** Final models for the association between weekly building-related symptoms and all variables (Model 4)

Variable factors	Eye irritation OR (95% CI)	General symptoms^*l*^ OR (95% CI)	Upper respiratory OR (95% CI)	Skin symptoms^*l*^ OR (95% CI)
	*N* = 2330	*N* = 2275	*N* = 2260	*N* = 2436
Personal				
Gender (female)	1.67 (1.23–2.28)^**^	1.80 (1.36–2.38)^**^	2.27 (1.48–3.48)^**^	3.12 (1.48–6.59)^**^
Age				
10–19	–	1.96 (0.19–19.71)	–	–
20–29	–	3.24 (1.68–6.23)^**^	–	–
30–39	–	2.16 (1.16–4.00)^*^	–	–
40–49	–	1.66 (0.89–3.11)	–	–
50–59	–	1.35 (0.71–2.58)	–	–
≥ 60	–	Ref.	–	–
*p* for trend	–	<0.001	–	–
Contact lens use	1.59 (1.20–2.10)^**^	–	1.73 (1.16–2.57)^**^	–
Pet ownership (Cat)			–	2.86 (1.28–6.43)^*^
Work environment				
No. of people in office^*a*^	1.19 (1.03–1.39)^*^	–	–	–
Work station				
Floor carpet (with)	1.44 (1.07–1.95)^*^	1.53 (1.15–2.05)^**^	1.74 (1.10–2.75)^*^	–
Reflection or glare in vision^*b*^	1.25 (1.07–1.45)^**^	1.32 (1.13–1.55)^**^	–	–
Chair comfort^*c*^	1.37 (1.13–1.67)^**^	1.35 (1.11–1.63)^**^	1.74 (1.34–2.27)^**^	–
Work with computer	5.51 (1.62–18.73)^**^	–		
Use of odorous chemicals^*d*^	1.11 (1.02–1.21)^*^	–	–	–
Change in workplace^*e*^				
Painted wall			4.72 (1.57–14.22)^**^	
Workplace conditions in last 4 weeks^*f*^				
Too little air movement	–	1.37 (1.21–1.56)^**^	1.26 (1.05–1.50)^*^	–
Varying room temperature	1.23 (1.07–1.42)^**^	–	–	–
Too cold	–	1.45 (1.21–1.73)^**^	–	–
Air too humid	–	1.20 (1.02–1.43)^*^	–	–
Air too dry	1.46 (1.24–1.72)^**^	–	1.76 (1.44–2.14)^**^	2.71 (2.10–3.51)^**^
Static electricity	–	–	–	–
Noise	1.29 (1.05–1.59)^*^	1.54 (1.25–1.88)^**^	1.41 (1.09–1.82)^**^	–
Dust and dirt	–	–	1.26 (1.00–1.58)^*^	–
Tobacco smoke odor	1.21 (1.02–1.43)^*^	–	–	–
Unpleasant chemical odor	–	–	1.96 (1.24–3.08)^**^	2.60 (1.63–4.15)^**^
Unpleasant other odor^*g*^	1.22 (1.02–1.45)^*^	1.35 (1.14–1.61)^**^	–	
Job stressors				
Amount of work^*h*^	1.34 (1.19–1.51)^**^	1.38 (1.23–1.55)^**^	1.25 (1.06–1.46)^**^	
Mental workload^*h*^	–	–		
Physical overload^*i*^	0.70 (0.59–0.83)^**^	–	–	
Interpersonal conflict^*h*^	–	1.42 (1.21–1.65)^**^	–	–
Job control^*h*^	–	0.80 (0.70–0.92)^**^	–	–
Skill utilization^*j*^	–	–	–	–
Job suitability^*k*^	–		–	
Work satisfaction^*k*^	–	0.76 (0.66–0.88)^**^	–	–

Values are expressed as adjusted odds ratio (95% CI) for participants with complete data. Personal factors, work environment, workplace conditions in the last 4 weeks, and job stressors with *p* < 0.2 in univariate analyse are included in multivariate stepwise logistic regression analysis (forward, Wald). Ref. = referent. Significant at ^*^ *p* < 0.05, ^**^ *p* < 0.01. Horizontal lines are expressed as the variable included in models

^*a*^ Number of people working in the room in which workstation of respondent is located. Six levels of response are 1) 1 person, 2) 2–3 persons, 3) 4–7 persons, 4) 8–20 persons, 5) 21–50 persons, and 6) ≥ 51 persons

^*b*^ Five levels of response are 1) rarely, 2) occasionally, 3) sometimes, 4) fairly often, and 5) very often

^*c*^ Four levels of response are 1) very comfortable, 2) reasonably comfortable, 3) somewhat uncomfortable, and 4) very uncomfortable

^*d*^ Five levels of response are 1) never, 2) less than 3 times/week, 3) 3–4 times a week, 4) about once a week, and 5) several times a day; with cleanser, glue, correction fluid, or other odorous chemicals

^*e*^ Change taken place within five meters of workstation in last 3 months

^*f*^ Four levels of response are 1) never, 2) 1–3 days, 3) 1–3 days per week, and 4) every or almost every workday

^*g*^ For example, body odor, food odor, or perfume

^*h*^ Five levels of response are 1) less/low, 2) somewhat less/low, 3) medium, 4) somewhat more/high, and 5) more/high

^*i*^ Four levels of response are 1) somewhat less/low, 2) medium, 3) somewhat more/high, and 4) more/high

^*j*^ Four levels of response are 1) less/low, 2) somewhat less/low, 3) medium, and 4) somewhat more/high

^*k*^ Four levels of response are 1) less/low, 2) somewhat less/low, 3) medium, and 4) more/high

^*l*^ For ensuring goodness of fit in the model, the variable highly correlated with other variable was excluded from the model, which variables were job suitability in general symptoms and unpleasant other odor in skin symptoms

#### Personal factors

All BRSs were significantly increased in females (Tables 3 and 6). A younger age (20–29 years, followed by 30–39 years) significantly increased the possibility of reported general symptoms. Current smoking was not related to BRSs (Table 3). Contact lens use was significantly related to eye irritation and upper respiratory symptoms. Owning a cat as a pet was significantly associated with skin symptoms (Table 6).

#### Job stressors

As shown in Table 3, multivariate analyses revealed that many stressors, such as excessive work, high mental workload, strong interpersonal conflict, low job control, and low work satisfaction, were significantly associated with general symptoms (Model 1). Even after being adjusted for additional variables of work environment and workplace conditions in the final multivariate model (Model 4), these associations persisted, apart from the association with high mental workload (Table 6). Excessive work, high mental workload, low physical overload, strong interpersonal conflict, and low skill utilization were significantly associated with eye irritation, and excessive work and strong interpersonal conflict significantly increased the reporting of upper respiratory symptoms (Table 3, Model 1). The association with strong interpersonal conflict was also significant in skin symptoms. However, the associations did not persist after adjusting for other variables in the final model (Model 4) apart from the association with excessive work and low physical overload (Table 6).

#### Work environment

No significant association between the office equipment and BRSs was observed (Table 4, Model 2). In the final model (Table 6), a crowded workplace was significantly associated with eye irritation. Carpeting and uncomfortable seating were significantly associated with eye irritation, general symptoms, and upper respiratory symptoms. Often a reflection or glare was significantly associated with eye irritation and general symptoms. The increased use of odorous chemicals was significantly associated with eye irritation. The association between eye irritation and working on the computer was strongly significant (OR, 5.51; 95% CI, 1.62–18.73) (Model 4). The association between upper respiratory symptoms and recently painted wall (in the last 3 months) in the workplace within five meters of a workstation was also strongly significant (OR, 4.72; 95% CI, 1.57–14.22) (Model 4).

#### Workplace conditions

In the associations between the workplace conditions in the last 4 weeks and BRSs, multivariate analyses revealed that all symptoms were significantly associated with air-conditioning factors and unpleasant odors (e.g., body, food, or chemical odors) (Tables 5 and 6). In the final model (Model 4), dryness of air was significantly associated with eye irritation, upper respiratory symptoms, and skin symptoms (Table 6). Mucosal dryness involves these symptoms. The association with skin symptoms was strongest (OR, 2.71; 95% CI, 2.10–3.51). However, general symptoms were significantly associated with humidity and cold. The association between unpleasant chemical odors (e.g., cleanser, glue, correction fluid, or other odorous chemicals) and skin symptoms was the strongest in indoor pollutant factors (OR, 2.60; 95% CI, 1.63–4.15). Noise was significantly associated with eye irritation, general symptoms, and upper respiratory symptoms.

### Seasonal comparisons of prevalence and risk factors of BRSs

In winter, of the original 489 offices, 320 offices provided office worker responses, and questionnaire responses were obtained from 3335 office workers [28]. In summer, 3024 questionnaire responses from 309 offices that were provided from its office workers were obtained. The number of offices providing questionnaire responses from office workers in both winter and summer was 246.

The comparison of the prevalence of weekly BRSs between winter and summer is shown in Table 7. Univariate analyses (based on a chi-square test) comparing personal factors between winter and summer indicated statistically differences (*p* < 0.2) across age, gender, and job. Adjusted OR was calculated by the Mantel-Haenszel method and adjusted for these personal factors. The prevalence of three general symptoms (“Tension, irritability, or nervousness,” “feeling depressed,” and “unusual tiredness, fatigue, or drowsiness”) in summer was significantly higher than those in winter. The prevalence of the symptoms of “tired or strained eyes” in summer was also higher than that in winter. However, the prevalence of symptoms of “sore or dry throat” and symptoms of “dry or flushed facial skin” in summer was lower than that in winter. In addition, for comparison between winter and summer using potential risk factors (personal factors, work environment, workplace conditions in the last 4 weeks, and job stressors), logistic regression analyses were conducted adding the seasonal factor (summer and winter) to the combined data of summer and winter using the same method as that used in Model 4. The adjusted ORs (summer) of weekly BRSs in terms of eye irritation, general symptoms, upper respiratory symptoms, and skin symptoms were 2.05 (95% CI, 1.66–2.54; *p* < 0.001), 1.64 (95% CI, 1.34–2.01; *p* < 0.001), 1.06 (95% CI, 0.79–1.42; *p* = 0.699), and 0.70 (95% CI, 0.47–1.05; *p* = 0.084), respectively.

**Table 7:** Comparison of prevalence of weekly building-related symptoms between winter and summer

Symptoms	Prevalence ^*a*^ (%)		Summer (vs. winter)	
	Winter	Summer	Crude OR (95% CI)	Adjusted OR^b^ (95% CI)
Tension, irritability, or nervousness	8.77	11.58	1.36 (1.15–1.61)^**^	1.30 (1.09–1.55)^**^
Tired or strained eyes	7.99	10.21	1.31 (1.10–1.57)^**^	1.27 (1.05–1.53)^*^
Dry, itching, or irritated eyes	7.15	7.52	1.06 (0.87–1.28)	1.01 (0.82–1.23)
Feeling depressed	5.12	6.83	1.36 (1.10–1.68)^**^	1.31 (1.05–1.63)^*^
Unusual tiredness, fatigue, or drowsiness	4.71	6.39	1.38 (1.11–1.72)^**^	1.36 (1.08–1.70)^**^
Sore or dry throat	5.69	3.70	0.64 (0.50–0.81)^**^	0.56 (0.43–0.72)^**^
Headache	2.35	2.65	1.13 (0.82–1.56)	1.06 (0.76–1.47)
Difficulty remembering things or concentration	1.45	2.19	1.52 (1.03–2.23)^*^	1.46 (0.98–2.17)
Sneezing	1.82	1.84	1.01 (0.69–1.47)	0.97 (0.65–1.43)
Cough	1.55	1.24	0.80 (0.52–1.23)	0.73 (0.47–1.14)
Dry or flushed facial skin	3.18	1.21	0.37 (0.25–0.55)^**^	0.33 (0.22–0.49)^**^
Stuffy or runny nose, or sinus congestion	1.71	1.18	0.68 (0.44–1.05)	0.67 (0.43–1.04)
Hands dry, Itching, red skin	1.43	1.11	0.78 (0.49–1.22)	0.68 (0.42–1.08)
Dizziness or lightheadedness	0.74	1.09	1.48 (0.87–2.53)	1.38 (0.80–2.36)
Nausea or upset stomach	0.86	1.09	1.27 (0.76–2.11)	1.10 (0.65–1.84)
Scaling/itching scalp or ears	1.01	0.78	0.77 (0.45–1.31)	0.68 (0.39–1.16)
Shortness of breath	0.27	0.50	1.84 (0.80–4.20)	1.58 (0.69–3.64)
Chest tightness	0.52	0.50	0.97 (0.48–1.95)	0.84 (0.40–1.75)
Wheezing	0.21	0.23	1.10 (0.39–3.14)	1.14 (0.38–3.40)

^*a*^ A participant experienced the symptom at least 1 day per week in the last 4 weeks that improved when the participant was away from the building. Significant at ^*^ *p* < 0.05, ^**^ *p* < 0.01

^*b*^ Mantel-Haenszel test was carried out to estimate the adjusted odds ratio (OR) and the 95% confidence interval (95% CI) for the BRSs with/without adjustment for potential confounders, including age, gender, and job, which showed *p* < 0.2 in univariate analyses. Tarone test showed *p* > 0.05 in all symptoms

Significant risk factors associated with weekly BRSs in the final model in winter and summer are shown in Table 8. Excessive work was common risk factor of eye irritation and general symptoms in both seasons. Strong interpersonal conflict and low work satisfaction were also common risk factors for general symptoms.

**Table 8:** Significant risk factors associated with weekly building-related symptoms in final model in winter (*N* = 3335) and summer (*N* = 3024)

Symptoms and seasons	Significant risk factors		
	Job stressor	Work environment	Indoor air quality
Eye irritation			
Winter	• Excessive work • Adequate skill utilization	• Carpeting • Poor lighting • Uncomfortable seating • Often use of odorous chemicals^a^	• Too cold • Too dry • Strong static electricity • Airflow from air conditioner • Dust and dirt
Summer	• Excessive work • Low physical overload	• Crowded workplace • Carpeting • Often reflection or glare in vision • Uncomfortable seating • Computer work • Often use of odorous chemicals^a^	• Varying room temperature • Too dry • Noise • Tobacco smoke odor • Unpleasant other odor^b^
General symptoms			
Winter	• Excessive work • High mental workload • Strong interpersonal conflict • Low job suitability • Low work satisfaction	• Crowded workplace	• Too little air movement • Varying room temperature • Too cold • Too dry • Noise • Dust and dirt • Unpleasant other odor^b^
Summer	• Excessive work • Strong interpersonal conflict • Low job control • Low work satisfaction	• Carpeting • Reflection or glare in vision • Uncomfortable seating	• Too little air movement • Too cold • Too humid • Noise • Unpleasant other odor^b^
Upper respiratory			
Winter	• Strong interpersonal conflict	• Crowded workplace • Installation of bubble jet printer	• Too dry • Dust and dirt • Unpleasant other odor^b^
Summer	• Excessive work	• Carpeting • Uncomfortable seating • Recent painted wall	• Too little air movement • Too dry • Noise • Dust and dirt • Unpleasant chemical odor
Skin symptoms			
Winter	• Low work satisfaction		• Varying room temperature • Too dry • Noise • Airflow from air conditioner • Dust and dirt
Summer			• Too dry • Unpleasant chemical odor

^a^ Cleaning substance, adhesives, correction liquid, or odor products, etc

^b^ Body odor, food odor, or perfume, etc

In the work environment, carpeting, uncomfortable seating, and increased use of odorous chemicals were common risk factors for eye irritation in both seasons. Poor lighting was significant risk factor of eye irritation in winter but often reflection or glare was a risk factor in summer. Carpeting and uncomfortable seating were significant risk factors for general and upper respiratory symptoms in summer but such factors were not significant in winter. Particularly, a recently painted wall was a significant risk factor for upper respiratory symptoms in summer and a crowded workplace was a significant risk factor for general and upper respiratory symptoms in winter.

In the risk factors related to indoor air quality, dryness of air was a common risk factor for BRSs in both seasons, but humidity was the only significant risk factor for general symptoms in summer. In particular, unpleasant chemical odors were a significant risk factor for upper respiratory and skin symptoms in summer but not in winter. Noise, dust and dirt, and unpleasant odors (e.g., body odors, food odors, or perfume) were significant risk factors for some BRSs in both seasons.

## Discussion

The U.S. Environmental Protection Agency (USEPA) conducted a Building Assessment Survey Evaluation (BASE) study in 100 office buildings in the United States, the sites of work of 4326 office workers, in the 1990s [40]. The questionnaire used in our survey comprised the USEPA questionnaire [33] used in the BASE study. Therefore, the prevalence estimated in our study can be exactly compared with the prevalence estimated in the BASE study. The BASE study identified the three most prevalent BRSs as “tired or strained eyes” (22%), “dry, itching, or irritated eyes” (19%), and “pain or stiffness in the back, shoulders, or neck” (17%). The lowest prevalence was associated with “shortness of breath and wheezing” (2%). In our study, the three most prevalent BRSs during winter were “tension, irritability, or nervousness” (8.8%), “tired or strained eyes” (8.0%), and “dry, itching, or irritated eyes” (7.1%). The prevalence of “tension, irritability, or nervousness,” “tired or strained eyes,” and “dry, itching, or irritated eyes” during summer were 11.6%, 10.2%, and 7.5%, respectively. The lowest prevalence was “wheezing” (0.2%) in both seasons. In both BASE and our studies, the prevalence of BRSs followed approximately the same rank ordering. Compared with the BASE study, overall prevalence was lower in our studies. However, the prevalence of “feeling depressed” in both seasons of our studies was higher than that of the BASE study. The prevalence of “tension, irritability, or nervousness” in summer, and that of “sore or dry throat” in winter, was close to the prevalence of the BASE study. Prevalence obtained from the present study were also substantially lower than those of a recent study [10]. In Japan, the Law for Maintenance of Sanitation in Buildings was enacted in 1970 and the Building Sanitation Management Standards were also established in 1970. Those standards might be effective for prevention of BRSs.

BRSs were common, with approximately one in four persons having at least one BRS. Their prevalence in summer (27.8%) was slightly higher than that in winter (24.9%) [28]. The prevalence of eye irritation, general symptoms, upper respiratory symptoms, lower respiratory symptoms, and skin symptoms was 12.1%, 14.4%, 8.9%, 0.8%, and 4.5%, respectively, in the study in winter [28] and 14.1%, 18.3%, 6.7%, 0.9%, and 2.2%, respectively, in summer. Overall, general symptoms therefore had the highest prevalence, followed by eye irritation and upper respiratory symptoms in both winter and summer. Lower respiratory symptoms had the lowest prevalence in both seasons.

Regarding the risk factors of BRSs, dryness of air was an important significant risk factor in both seasons in this study. This may influence the high prevalence of BRSs related to mucosal dryness. In our study, the prevalence of “dry, itching, or irritated eyes” was high, followed “tension, irritability, or nervousness” and “tired or strained eyes” in both seasons. Several studies have reported that the dryness of nose, throat, eyes, and skin was improved by adequate humidification [41–44]. Low humidity was associated with increased eye irritation and the alteration of the precorneal tear film (PTF) [26]. A visual display unit (VDU) can be used for computer work, as a monitor may exacerbate these effects. Our study indicated that computer work had a strongly significant association with eye irritation in summer.

Our study indicated that humidity was significantly associated with general symptoms in summer. Although summer in Japan is hot and humid, a campaign named Cool Biz has been implemented by the Japanese government since 2005, recommending raising the set points during summer to 28 °C and wearing lighter clothing [45]. At high temperatures, the degree of discomfort can be heavily influenced by humidity level [46]. Air-conditioning system with humidity control could create a comfortable work environment, even at temperatures as high as 30 °C, as compared to temperature control alone [47]. Adequate indoor humidity control will reduce general symptoms in hot and humid climate regions.

Recently painted walls and unpleasant chemical odors in summer were significantly associated with BRSs. Those effects are likely to be found in summer, as concentrations of specific volatile organic compounds may be especially elevated in a hot season. Higher indoor concentrations of aromatic and aliphatic hydrocarbons were found more often during winter in existing buildings, while the concentrations of carbonyl compounds, organophosphorus compounds, ammonia, and ozone, which include strong irritants to the upper respiratory tract and skin, were higher during summer [29–31]. Although a moderate association between upper respiratory symptoms, dry throat, and irritability related to buildings and concentrations of total volatile organic compound in the workplace have been suggested [48], studies reported that no consistent association between BRSs and indoor exposure levels of individual volatile organic compound or total volatile organic compound were found [49, 50]. No measurements of indoor pollutants were conducted in the present study. Future observational research during a hot season would provide valuable information for understanding those effects.

Noise, dust and dirt were significant risk factors for some BRSs in both seasons. The associations have been reported in the previous studies [10, 16, 51]. The presence of acoustical insulation and/or sound absorption materials increased overall building comfort and cleaning the work area in the morning before a workday began decreased both BRSs and overall building discomfort, compared to cleaning it in the evening after work [10]. However, further research is needed to clarify the reported relationships. In particular, noise was significantly associated with general symptoms, eye irritation, and upper respiratory symptoms during summer in the present study. Noise can involve general symptoms but may not be a cause of mucosal eye irritation or mucosal upper respiratory dysfunction. Persons with a high level of general symptom may be more sensitive to eye and upper respiratory mucosal dysfunction, and such dysfunction could trigger more general symptoms. Therefore, eye irritation and upper respiratory symptoms might be induced as a common sense with general symptoms. BRS is known as one of the medically unexplained symptoms including conditions such as multiple chemical sensitivity, chronic fatigue syndrome, fibromyalgia, and the like. BRS is characterized by subjective responses to nonspecific conditions associated with the use of a building and due to causes resulting from the complex interaction of several factors, such as exposure to indoor chemical and biological pollutants, indoor physical conditions, exposure to occupational stress, and individual susceptibility [52]. Investigation of involvement of psychosocial factors, including personality traits, personal circumstances, and individual perceptions is required.

General symptoms, including “tension, irritability, or nervousness,” “feeling depressed,” “unusual tiredness, fatigue, or drowsiness,” and so forth were significantly associated with excessive work, strong interpersonal conflict, and low work satisfaction in both seasons. A reduction in psychological distress related to work throughout the year is needed.

Our study had several limitations. We used a cross-sectional study design. The cross-sectional nature of the study limits any causal inferences and may be subject to a recall bias. Several environmental reports from respondents are subjective, and the resulting inaccuracies may have resulted in a bias. This is also true of the subjective, self-reported health outcome assessments used in this study. The office managers selected all office workers when 10 or less office workers in their office met our selection criteria, but otherwise, managers arbitrarily selected 15 office workers at a maximum that met the criteria. The proportion of participants that worked in offices with no more than 20 employees was 71.0% (72.6% in winter). Although office workers who met the exclusion criteria will have been included in that number, we cannot exclude selection bias. Thus, it is probably less likely that office workers with symptoms rather than those without symptoms tended to be selected as participants by the managers, and vice versa. The prevalence of BRSs obtained in this study would include such uncertainty. However, it is probably less likely that the selection bias severely influenced the strength of the associations between BRSs and the possible risk factors as well as seasonal comparisons of prevalence and risk factors of BRSs between winter and summer. Office workers suffering from BRSs may avoid working overtime, which may have underestimated the prevalence and risks of extensive overtime. Finally, we analyzed mass variable factors that could introduce a systematic statistical bias. We therefore performed a number of statistical analyses (four models, two stepwise procedures of Wald and likelihood ratio), and the results were similar for the different models used; thus, it is less likely that the analysis results of risk factors were affected by a particular statistical model or the large number of statistical tests performed.

## Conclusions

BRSs were common in this nationwide survey, with approximately one in four persons having at least one BRS in both winter and summer. Analyses suggested that adequate humidity control and managing job stress throughout the year will reduce BRSs. Physical risk factors such as recently painted walls and unpleasant chemical odors in summer and noise, dust and dirt, and unpleasant odors including body or food odors were significant risk factors of some BRSs in both the seasons. Improvements of the physical environmental qualities in the office environment are important. Moreover, the prevention of BRSs should not only depend on physical measures but also involve comprehensive interventions to improve psychosocial well-being and mental health in office workers. Our results suggest intervention points of physical and psychosocial environment for office managers and health professionals.

## Additional file

Additional file 1: Table S1. Univariate analysis for the association with weekly building-related symptoms. **Table S2** Number of cases by variable factors for weekly building-related symptoms. (DOCX 59 kb)
