# Supplementary material for: Evaluating prevalence and risk factors of building-related symptoms among office workers: Seasonal characteristics of symptoms and psychosocial and physical environmental factors
Source: Environ Health Prev Med. 2017 Apr 12;22:38. doi: 10.1186/s12199-017-0645-4 (PMC5664800; doi:10.1186/s12199-017-0645-4)
Supplement: Additional file 1: Table S1. — Univariate analysis for the association with weekly building-related symptoms. Table S2 Number of cases by variable factors for weekly building-related symptoms. (DOCX 59 kb) [file 12199_2017_645_MOESM1_ESM.docx]

Online supporting information for the following article published in *Environmental Health and Preventive Medicine*

**Evaluating prevalence and risk factors of building-related symptoms among office workers: Seasonal characteristics of symptoms and psychosocial and physical environmental factors**

Kenichi Azuma^1^, Koichi Ikeda^2^, Naoki Kagi^3^, U Yanagi^4^, Haruki Osawa^5^

*1 Corresponding Author: Department of Environmental Medicine and Behavioral Science, Kindai University Faculty of Medicine, Osakasayama, Osaka, Japan*

*2 Department of Architecture, College of Science and Technology, Nihon University, Chiyoda-ku, Tokyo, Japan*

*3 Department of Mechanical and Environmental Informatics, Graduate School of Information Science and Engineering, Tokyo Institute of Technology, Meguro-ku, Tokyo, Japan*

*4 Department of Architecture, School of Architecture, Kogakuin University, Shinjuku-ku, Tokyo, Japan*

*5 National Institute of Public Health, Wako, Saitama, Japan*

**Table S1.** Univariate analysis for the association with weekly building-related symptoms.

| Variable factors | Eye irritation  OR (95% CI) | General symptoms  OR (95% CI) | Upper respiratory  OR (95% CI) | Skin symptoms  OR (95% CI) |
| --- | --- | --- | --- | --- |
| Personal |  |  |  |  |
| Gender (female) | 3.52 (2.82–4.40)^**^ | 2.73 (2.23–3.33)^**^ | 4.00 (2.89–5.54)^**^ | 6.68 (3.61–12.36)^**^ |
| Age |  |  |  |  |
| 10–19 | 3.47 (0.39–31.23) | 2.14 (0.24–18.92) | 5.95 (0.64–55.27) | – |
| 20–29 | 5.23 (3.08–8.88)^**^ | 4.45 (2.83–6.99)^**^ | 3.89 (1.94–7.79)^**^ | 1.55 (0.62–3.90) |
| 30–39 | 3.28 (1.99–5.42)^**^ | 3.04 (2.00–4.62)^**^ | 2.56 (1.33–4.93)^**^ | 1.12 (0.49–2.59) |
| 40–49 | 2.59 (1.55–4.31)^**^ | 2.12 (1.38–3.25)^**^ | 1.62 (0.82–3.22) | 0.78 (0.32–1.89) |
| 50–59 | 2.69 (1.60–4.52)^**^ | 1.91 (1.22–2.97)^**^ | 1.79 (0.89–3.60) | 0.73 (0.29–1.88) |
| ≥60 | Ref. | Ref. | Ref. | Ref. |
| *p* for trend | < 0.001 | < 0.001 | < 0.001 | 0.541 |
| Job categories |  |  |  |  |
| Managerial | Ref. | Ref. | Ref. | Ref. |
| Professional | 0.57 (0.27–1.23) | 0.97 (0.53–1.75) | 1.28 (0.42–3.91) | 0.94 (0.11–8.10) |
| Technical | 044 (0.26–0.75)^**^ | 0.85 (0.56–1.27) | 1.45 (0.69–3.04) | 0.63 (0.12–3.24) |
| Sales | 0.74 (0.46–1.19) | 1.21 (0.82–1.80) | 1.32 (0.60–2.91) | 1.46 (0.39–5.46) |
| Planning/administrative | 2.63 (1.96–3.53)^**^ | 2.61 (1.98–3.46)^**^ | 4.83 (2.80–8.33)^**^ | 5.05 (1.99–12.79)^**^ |
| Secretarial/clerical | 5.73 (0.94–34.92) | 1.79 (0.20–16.21) | 19.13 (3.25–112.68)^**^ | – |
| Other | 0.40 (0.17–0.94)^*^ | 1.08 (0.60–1.93) | 3.02 (1.29–7.07)^*^ | 4.63 (1.32–16.21)^*^ |
| Smoking |  |  |  |  |
| Never | Ref. | Ref. | Ref. | Ref. |
| Former | 0.51 (0.38–0.68)^**^ | 0.55 (0.43–0.72)^**^ | 0.57 (0.38–0.85)^**^ | 0.66 (0.35–1.25) |
| Current/sometime | 0.64 (0.34–1.23) | 0.50 (0.26–0.96)^*^ | 0.74 (0.32–1.75) | 0.37 (0.05–2.71) |
| Current/everyday | 0.51 (0.40–0.67)^**^ | 0.63 (0.49–0.79)^**^ | 0.48 (0.33–0.70)^**^ | 0.55 (0.30–1.04) |
| Contact lens use | 2.58 (2.05–3.25)^**^ | 2.04 (1.64–2.54)^**^ | 2.35 (1.72–3.26)^**^ | 2.16 (1.27–3.65)^**^ |
| Pet ownership at home |  |  |  |  |
| Dog | 1.07 (0.80–1.42) | 1.02 (0.79–1.33) | 0.87 (0.57–1.34) | 0.62 (0.28–1.37) |
| Cat | 0.85 (0.56–1.29) | 1.01 (0.70–1.44) | 1.82 (1.15–2.89)^*^ | 3.41 (1.85–6.28)^**^ |
| Work environment |  |  |  |  |
| No. of people in office*^a^* | 1.45 (1.28–1.63)^**^ | 1.35 (1.20–1.50)^**^ | 1.29 (1.09–1.53)^**^ | 1.38 (1.04–1.82)^*^ |
| Work station |  |  |  |  |
| Floor carpet (with) | 1.63 (1.27–2.09)^**^ | 1.45 (1.16–1.81)^**^ | 1.73 (1.21–2.48)^**^ | 2.30 (1.19–4.43)^*^ |
| Lighting*^b^* | 0.81 (0.63–1.03) | 0.81 (0.65–1.02) | 0.57 (0.42–0.79)^**^ | 0.58 (0.35–0.96)^*^ |
| Reflection or glare in vision*^c^* | 1.73 (1.53–1.96)^**^ | 1.71 (1.52–1.92)^**^ | 1.73 (1.48–2.03)^**^ | 2.01 (1.60–2.53)^**^ |
| Table comfort*^d^* | 1.67 (1.42–1.96)^**^ | 2.04 (1.75–2.37)^**^ | 1.95 (1.57–2.43)^**^ | 2.36 (1.67–3.32)^**^ |
| Chair comfort*^d^* | 1.77 (1.52–2.07)^**^ | 2.00 (1.73–2.32)^**^ | 2.36 (1.92–2.91)^**^ | 2.24 (1.60–3.14)^**^ |
| Work with computer | 6.09 (2.99–12.41)^**^ | 1.99 (1.33–2.98)^**^ | 1.27 (0.73–2.23) | 0.76 (0.36–1.62) |
| Use of odorous chemicals*^e^* | 1.22 (1.14–1.30)^**^ | 1.23 (1.15–1.31)^**^ | 1.22 (1.11–1.34)^**^ | 1.23 (1.05–1.44)^**^ |
| Change in workplace*^f^* |  |  |  |  |
| New carpeting | 1.93 (0.97–3.84) | 0.70 (0.30–1.67) | 2.55 (1.12–5.77)^*^ | 1.88 (0.45–7.92) |
| Painted wall | 1.04 (0.43–2.48) | 1.24 (0.56–2.72) | 2.55 (1.05–6.15)^*^ | 1.03 (0.14–7.62) |
| New furniture | 1.42 (0.88–2.29) | 1.25 (0.78–2.00) | 1.75 (0.94–3.25) | 1.19 (0.37–3.87) |
| New partitions | 1.08 (0.50–2.30) | 1.12 (0.56–2.26) | 1.52 (0.60–3.86) | 1.74 (0.41–7.30) |
| New wall covering | 1.89 (0.98–3.63) | 0.94 (0.44–2.03) | 2.41 (1.06–5.43)^*^ | 0.84 (0.11–6.15) |
| Water damage | 1.27 (0.76–2.10) | 1.24 (0.77–2.01) | 1.19 (0.57–2.49) | 2.61 (1.10–6.19)^*^ |
| Equipment/installation |  |  |  |  |
| Laser printer*^g^* | 1.34 (1.07–1.69)^*^ | 1.34 (1.08–1.65)^**^ | 1.23 (0.89–1.70) | 1.12 (0.65–1.94) |
| Bubble jet printer*^g^* | 1.17 (0.93–1.48) | 1.25 (1.01–1.55)^*^ | 0.91 (0.64–1.28) | 1.97 (1.18–3.30)^*^ |
| Copier*^g^* | 1.28 (1.02–1.61)^*^ | 1.41 (1.14–1.73)^**^ | 1.35 (0.98–1.86) | 1.70 (1.01–2.84)^*^ |
| Exterior window*^g^* | 0.87 (0.70–1.09) | 0.85 (0.69–1.04) | 0.77 (0.56–1.06) | 1.10 (0.66–1.84) |
| Door*^g^* | 0.75 (0.59–0.96)^*^ | 0.95 (0.77–1.19) | 1.02 (0.73–1.42) | 1.28 (0.76–2.179 |
| Fragrance*^h^* | 0.90 (0.61–1.32) | 0.83 (0.58–1.20) | 1.24 (0.74–2.05) | 1.75 (0.85–3.58) |
| Air freshener*^h^* | 1.18 (0.87–1.61) | 1.09 (0.81–1.46) | 1.48 (0.98–2.23) | 1.34 (0.67–2.65) |
| Repellent*^h^* | 1.11 (0.79–1.55) | 1.00 (0.73–1.38) | 1.68 (1.10–2.57)^*^ | 1.71 (0.86–3.40) |
| Workplace conditions in last four weeks*^i^* |  |  |  |  |
| Too much air movement | 1.45 (1.12–1.87)^**^ | 1.87 (1.47–2.38)^**^ | 2.11 (1.60–2.79)^**^ | 1.86 (1.22–2.84)^**^ |
| Too little air movement | 1.77 (1.61–1.95)^**^ | 2.18 (1.98–2.39)^**^ | 2.22 (1.96–2.51)^**^ | 2.14 (1.76–2.60)^**^ |
| Too hot | 1.50 (1.36–1.66)^**^ | 1.59 (1.45–1.74)^**^ | 1.68 (1.48–1.92)^**^ | 1.66 (1.34–2.05)^**^ |
| Varying room temperature | 1.83 (1.64–2.03)^**^ | 1.85 (1.67–2.05)^**^ | 2.02 (1.76–2.32)^**^ | 2.28 (1.84–2.82)^**^ |
| Too cold | 1.67 (1.45–1.92)^**^ | 2.03 (1.77–2.33)^**^ | 2.02 (1.71–2.38)^**^ | 1.94 (1.51–2.50)^**^ |
| Air too humid | 1.79 (1.57–2.03)^**^ | 1.93 (1.71–2.19)^**^ | 1.66 (1.41–1.97)^**^ | 1.76 (1.37–2.26)^**^ |
| Air too dry | 2.38 (2.09–2.71)^**^ | 2.37 (2.08–2.70)^**^ | 2.85 (2.45–3.30)^**^ | 3.26 (2.65–4.01)^**^ |
| Static electricity | 2.44 (1.91–3.13)^**^ | 2.45 (1.90–3.17)^**^ | 2.34 (1.78–3.08)^**^ | 2.83 (2.03–3.95)^**^ |
| Noise | 1.83 (1.58–2.13)^**^ | 2.35 (2.02–2.75)^**^ | 2.23 (1.87–2.65)^**^ | 2.16 (1.69–2.77)^**^ |
| Airflow from air conditioner | 1.41 (1.27–1.57)^**^ | 1.50 (1.36–1.66)^**^ | 1.60 (1.40–1.83)^**^ | 1.49 (1.20–1.86)^**^ |
| Odors from air conditioner | 1.85 (1.58–2.17)^**^ | 2.00 (1.71–2.34)^**^ | 2.21 (1.84–2.64)^**^ | 2.24 (1.74–2.89)^**^ |
| Mold odor | 1.93 (1.61–2.30)^**^ | 2.12 (1.77–2.53)^**^ | 2.55 (2.10–3.10)^**^ | 2.19 (1.66–2.90)^**^ |
| Dust and dirt | 1.93 (1.70–2.19)^**^ | 2.16 (1.90–2.46)^**^ | 2.56 (2.21–2.97)^**^ | 2.27 (1.83–2.83)^**^ |
| Tobacco smoke odor | 1.65 (1.46–1.86)^**^ | 1.82 (1.61–2.05)^**^ | 1.87 (1.62–2.16)^**^ | 2.03 (1.63–2.52)^**^ |
| Unpleasant chemical odor | 1.77 (1.32–2.37)^**^ | 2.22 (1.66–2.98)^**^ | 3.10 (2.26–4.24)^**^ | 3.59 (2.57–5.03)^**^ |
| Unpleasant other odor*^j^* | 1.92 (1.69–2.18)^**^ | 2.40 (2.11–2.73)^**^ | 2.28 (1.97–2.65)^**^ | 2.60 (2.10–3.20)^**^ |
| Job stressors |  |  |  |  |
| Amount of work*^k^* | 1.19 (1.08–1.31)^**^ | 1.34 (1.23–1.46)^**^ | 1.18 (1.04–1.35)^*^ | 1.06 (0.85–1.33) |
| Mental workload*^k^* | 1.11 (1.00–1.24) | 1.22 (1.10–1.34)^**^ | 1.03 (0.88–1.20) | 1.03 (0.79–1.32) |
| Physical overload*^l^* | 0.62 (0.54–0.70)^**^ | 0.84 (0.76–0.93)^**^ | 0.80 (0.68–0.94)^**^ | 0.86 (0.66–1.12) |
| Interpersonal conflict*^k^* | 1.29 (1.15–1.45)^**^ | 2.00 (1.78–2.24)^**^ | 1.45 (1.23–1.71)^**^ | 1.67 (1.27–2.19)^**^ |
| Job control*^k^* | 0.82 (0.74–0.92)^**^ | 0.64 (0.57–0.70)^**^ | 0.77 (0.66–0.90)^**^ | 0.84 (0.65–1.07) |
| Skill utilization*^m^* | 0.71 (0.62–0.81)^**^ | 0.68 (0.60–0.77)^**^ | 0.62 (0.51–0.74)^**^ | 0.72 (0.53–0.99)^*^ |
| Job suitability*^n^* | 0.81 (0.72–0.90)^**^ | 0.69 (0.62–0.77)^**^ | 0.79 (0.67–0.93)^**^ | 0.97 (0.75–1.24) |
| Work satisfaction*^n^* | 0.79 (0.71–0.88)^**^ | 0.57 (0.51–0.63)^**^ | 0.69 (0.59–0.82)^**^ | 0.79 (0.61–1.02) |

Values are expressed as crude odds ratios (95% CI) for participants with complete data. Ref. = referent. Significant at ^*^ *p* < 0.05, ^**^ *p* < 0.01. Text in parentheses reflects case groups. *^a^* Number of people working in the room in which workstation of respondent is located. Six levels of response are 1) 1 person, 2) 2–3 persons, 3) 4–7 persons, 4) 8–20 persons, 5) 21–50 persons, and 6) ≥ 51 persons. *^b^* Five levels of response are 1) much too dim, 2) a little too dim, 3) Just right, 4) a little too bright, and 5) much too bright. *^c^* Five levels of response are 1) rarely, 2) occasionally, 3) sometimes, 4) fairly often, and 5) very often. *^d^* Four levels of response are 1) very comfortable, 2) reasonably comfortable, 3) somewhat uncomfortable, and 4) very uncomfortable. *^e^* Five levels of response are 1) never, 2) less than 3 times/week, 3) 3–4 times a week, 4) about once a week, and 5) several times a day; with cleanser, glue, correction fluid, or other odorous chemicals. *^f^* Change taken place within five meters of workstation in last three months. *^g^* Within two meters of workstation. *^h^* In workplace indoors. *^i^* Four levels of response are 1) never, 2) 1–3 days, 3) 1–3 days per week, and 4) every or almost every workday. *^j^* For example, body odor, food odor, or perfume. *^k^* Five levels of response are 1) less/low, 2) somewhat less/low, 3) medium, 4) somewhat more/high, and 5) more/high. *^l^* Four levels of response are 1) somewhat less/low, 2) medium, 3) somewhat more/high, and 4) more/high. *^m^* Four levels of response are 1) less/low, 2) somewhat less/low, 3) medium, and 4) somewhat more/high. *^n^* Four levels of response are 1) less/low, 2) somewhat less/low, 3) medium, and 4) more/high.

**Table S2.** Number of cases by variable factors for weekly building-related symptoms.

| Variable factors | Eye irritation  *n* (%) | General symptoms  *n* (%) | Upper respiratory  *n* (%) | Skin symptoms  *n* (%) |
| --- | --- | --- | --- | --- |
| Personal |  |  |  |  |
| Gender (female) | 251 (63.9) | 280 (57.1) | 123 (68.7) | 50 (79.4) |
| Age |  |  |  |  |
| 10–19 | 1 (0.3) | 1 (0.2) | 1 (0.6) | 0 (0.0) |
| 20–29 | 75 (19.2) | 92 (18.9) | 36 (20.3) | 11 (18.0) |
| 30–39 | 121 (30.9) | 162 (33.2) | 59 (33.3) | 19 (31.1) |
| 40–49 | 95 (24.3) | 117 (24.0) | 37 (20.9) | 13 (21.3) |
| 50–59 | 80 (20.5) | 87 (17.8) | 33 (18.6) | 10 (16.4) |
| ≥60 | 19 (4.9) | 29 (5.9) | 11 (6.2) | 8 (13.1) |
| Job categories |  |  |  |  |
| Managerial | 63 (16.2) | 73 (15.1) | 15 (8.4) | 5 (8.1) |
| Professional | 8 (2.1) | 15 (3.1) | 4 (2.2) | 1 (1.6) |
| Technical | 19 (4.9) | 40 (8.3) | 14 (7.8) | 2 (3.2) |
| Sales | 27 (6.9) | 47 (9.7) | 11 (6.1) | 4 (6.5) |
| Planning/administrative | 265 (67.9) | 292 (60.3) | 124 (69.3) | 45 (72.6) |
| Secretarial/clerical | 2 (0.5) | 1 (0.2) | 2 (1.1) | 0 (0.0) |
| Other | 6 (1.5) | 16 (3.3) | 9 (5.0) | 5 (8.1) |
| Smoking |  |  |  |  |
| Never | 224 (56.9) | 263 (53.6) | 104 (57.5) | 35 (55.6) |
| Former | 70 (17.8) | 91 (18.5) | 34 (18.8) | 13 (20.6) |
| Current/sometime | 11 (2.8) | 11 (2.2) | 6 (3.3) | 1 (1.6) |
| Current/everyday | 89 (22.6) | 126 (25.7) | 37 (20.4) | 14 (22.2) |
| Contact lens use | 142 (36.1) | 152 (31.1) | 65 (36.3) | 22 (34.9) |
| Pet ownership at home |  |  |  |  |
| Dog | 68 (17.3) | 81 (16.6) | 26 (14.4) | 7 (11.1) |
| Cat | 27 (7.0) | 40 (8.3) | 23 (13.0) | 14 (22.6) |
| Work environment |  |  |  |  |
| No. of people in office*^a^* |  |  |  |  |
| 1 person | 0 (0.0) | 0 (0.0) | 0 (0.0) | 0 (0.0) |
| 2–3 persons | 6 (1.5) | 11 (2.2) | 4 (2.2) | 1 (1.6) |
| 4–7 persons | 37 (9.4) | 63 (12.8) | 23 (12.8) | 7 (11.1) |
| 8–20 person | 195 (49.6) | 227 (46.2) | 81 (45.0) | 28 (44.4) |
| 21–50 persons | 126 (32.1) | 151 (30.8) | 62 (34.4) | 24 (38.1) |
| ≥ 51 persons | 29 (7.4) | 39 (7.9) | 10 (5.6) | 3 (4.8) |
| Work station |  |  |  |  |
| Floor carpet (with) | 300 (76.3) | 365 (74.2) | 141 (77.9) | 52 (82.5) |
| Lighting |  |  |  |  |
| Much too dim | 3 (0.8) | 5 (1.0) | 2 (1.1) | 0 (0.0) |
| A little too dim | 75 (19.0) | 96 (19.5) | 45 (24.9) | 20 (31.7) |
| Just right | 297 (75.4) | 362 (73.6) | 127 (70.2) | 38 (60.3) |
| A little too bright | 18 (4.6) | 28 (5.7) | 6 (3.3) | 5 (7.9) |
| Much too bright | 1 (0.3) | 1 (0.2) | 1 (0.6) | 0 (0.0) |
| Reflection or glare in vision |  |  |  |  |
| Rarely | 227 (57.6) | 287 (58.2) | 103 (56.9) | 31 (49.2) |
| Occasionally | 91 (23.1) | 120 (24.3) | 39 (21.5) | 11 (17.5) |
| Sometimes | 56 (14.2) | 64 (13.0) | 26 (14.4) | 16 (25.4) |
| Fairly often | 12 (3.0) | 16 (3.2) | 8 (4.4) | 3 (4.8) |
| Very often | 8 (2.0) | 6 (1.2) | 5 (2.8) | 2 (3.2) |
| Table comfort |  |  |  |  |
| Very comfortable | 30 (7.7) | 31 (6.5) | 15 (8.5) | 5 (8.2) |
| Reasonably comfortable | 238 (61.0) | 278 (58.3) | 95 (53.7) | 25 (41.0) |
| Somewhat uncomfortable | 96 (24.6) | 133 (27.9) | 51 (28.8) | 26 (42.6) |
| Very uncomfortable | 26 (6.7) | 35 (7.3) | 16 (9.0) | 5 (8.2) |
| Chair comfort |  |  |  |  |
| Very comfortable | 27 (7.0) | 33 (6.9) | 12 (6.9) | 5 (8.6) |
| Reasonably comfortable | 231 (59.5) | 265 (55.8) | 80 (46.0) | 25 (43.1) |
| Somewhat uncomfortable | 93 (24.0) | 131 (27.6) | 60 (34.5) | 20 (34.5) |
| Very uncomfortable | 37 (9.5) | 46 (9.7) | 22 (12.6) | 8 (13.8) |
| Work with computer | 386 (98.0) | 465 (94.3) | 167 (92.3) | 55 (87.3) |
| Use of odorous chemicals*^b^* |  |  |  |  |
| Never | 124 (32.0) | 149 (30.9) | 54 (31.0) | 21 (34.4) |
| Less than 3 times/week | 76 (19.6) | 101 (21.0) | 31 (17.8) | 11 (18.0) |
| 3–4times a week | 45 (11.6) | 64 (13.3) | 24 (13.8) | 3 (4.9) |
| About once a week | 55 (14.2) | 55 (11.4) | 25 (14.4) | 8 (13.1) |
| Several times a day | 87 (22.5) | 113 (23.4) | 40 (23.0) | 18 (29.5) |
| Change in workplace*^c^* |  |  |  |  |
| New carpeting | 11 (2.8) | 6 (1.2) | 7 (4.0) | 2 (3.2) |
| Painted wall | 6 (1.5) | 8 (1.6) | 6 (3.4) | 1 (1.6) |
| New furniture | 22 (5.6) | 23 (4.7) | 12 (6.7) | 3 (4.8) |
| New partitions | 8 (2.0) | 10 (2.1) | 5 (2.8) | 2 (3.2) |
| New wall covering | 12 (3.1) | 8 (1.6) | 7 (4.0) | 1 (1.6) |
| Water damage | 19 (4.9) | 22 (4.5) | 8 (4.5) | 6 (9.8) |
| Equipment/installation |  |  |  |  |
| Laser printer*^d^* | 134 (34.6) | 164 (34.3) | 58 (33.5) | 19 (31.1) |
| Bubble jet printer*^d^* | 125 (32.6) | 156 (33.0) | 48 (27.7) | 27 (45.0) |
| Copier*^d^* | 132 (34.0) | 169 (35.4) | 63 (35.8) | 25 (41.0) |
| Exterior window*^d^* | 145 (37.6) | 181 (37.7) | 62 (34.8) | 26 (42.6) |
| Door*^d^* | 97 (25.2) | 138 (29.0) | 53 (30.5) | 22 (35.5) |
| Fragrance*^e^* | 32 (8.2) | 37 (7.6) | 18 (10.1) | 9 (14.5) |
| Air refresher*^e^* | 56 (14.2) | 64 (13.1) | 30 (16.6) | 10 (15.9) |
| Repellent*^e^* | 45 (11.6) | 51 (10.5) | 28 (15.7) | 10 (16.7) |
| Workplace conditions in last four weeks |  |  |  |  |
| Too much air movement |  |  |  |  |
| Never | 370 (94.1) | 457 (92.9) | 161 (89.4) | 55 (88.7) |
| 1–3 days | 11 (2.8) | 17 (3.5) | 8 (4.4) | 4 (6.5) |
| 1–3 days per week | 7 (1.8) | 10 (2.0) | 7 (3.9) | 2 (3.2) |
| Every or almost every workday | 5 (1.3) | 9 (2.1) | 4 (2.2) | 1 (1.6) |
| Too little air movement |  |  |  |  |
| Never | 207 (52.7) | 244 (49.6) | 69 (38.3) | 19 (30.6) |
| 1–3 days | 73 (18.6) | 76 (15.4) | 31 (17.2) | 15 (24.2) |
| 1–3 days per week | 38 (9.7) | 57 (11.6) | 26 (14.4) | 12 (19.4) |
| Every or almost every workday | 75 (19.1) | 115 (23.4) | 54 (30.0) | 16 (25.8) |
| Too hot |  |  |  |  |
| Never | 160 (40.7) | 205 (41.7) | 66 (36.7) | 21 (33.9) |
| 1–3 days | 96 (24.4) | 108 (22.0) | 41 (22.8) | 15 (24.2) |
| 1–3 days per week | 81 (20.6) | 102 (20.7) | 34 (18.9) | 13 (21.0) |
| Every or almost every workday | 56 (14.2) | 77 (15.7) | 39 (21.7) | 13 (21.0) |
| Varying room temperature |  |  |  |  |
| Never | 198 (50.4) | 252 (51.2) | 78 (43.3) | 21 (33.9) |
| 1–3 days | 83 (21.1) | 107 (21.7) | 41 (22.8) | 16 (25.8) |
| 1–3 days per week | 68 (17.3) | 79 (16.1) | 31 (17.2) | 8 (12.9) |
| Every or almost every workday | 44 (11.2) | 54 (11.0) | 30 (16.7) | 17 (27.4) |
| Too cold |  |  |  |  |
| Never | 286 (72.8) | 354 (72.0) | 119 (66.1) | 41 (66.1) |
| 1–3 days | 62 (15.8) | 68 (13.8) | 29 (16.1) | 9 (14.5) |
| 1–3 days per week | 26 (6.6) | 40 (8.1) | 17 (9.4) | 5 (8.1) |
| Every or almost every workday | 19 (4.8) | 30 (6.1) | 15 (8.3) | 7 (11.3) |
| Air too humid |  |  |  |  |
| Never | 254 (64.60) | 320 (65.0) | 120 (66.7) | 40 (64.5) |
| 1–3 days | 78 (19.8) | 94 (19.1) | 31 (17.2) | 8 (12.9) |
| 1–3 days per week | 40 (10.2) | 45 (9.1) | 17 (9.4) | 9 (14.5) |
| Every or almost every workday | 21 (5.3) | 33 (6.7) | 12 (6.7) | 5 (8.1) |
| Air too dry |  |  |  |  |
| Never | 241 (61.3) | 323 (65.7) | 89 (49.4) | 20 (32.3) |
| 1–3 days | 73 (18.6) | 81 (16.5) | 32 (17.8) | 14 (22.6) |
| 1–3 days per week | 41 (10.4) | 45 (9.1) | 27 (15.0) | 12 (19.4) |
| Every or almost every workday | 38 (9.7) | 43 (8.7) | 32 (17.8) | 16 (25.8) |
| Static electricity |  |  |  |  |
| Never | 345 (87.8) | 438 (89.0) | 154 (85.6) | 50 (80.6) |
| 1–3 days | 27 (6.9) | 34 (6.9) | 14 (7.8) | 4 (6.5) |
| 1–3 days per week | 16 (4.1) | 13 (2.6) | 9 (5.0) | 5 (8.1) |
| Every or almost every workday | 5 (1.3) | 7 (1.4) | 3 (1.7) | 3 (4.8) |
| Noise |  |  |  |  |
| Never | 308 (78.4) | 368 (74.8) | 129 (71.7) | 42 (67.7) |
| 1–3 days | 49 (12.5) | 71 (14.4) | 24 (13.3) | 8 (12.9) |
| 1–3 days per week | 11 (2.8) | 18 (3.7) | 9 (5.0) | 6 (9.7) |
| Every or almost every workday | 25 (4.4) | 35 (7.1) | 18 (10.0) | 6 (9.7) |
| Airflow from air conditioner |  |  |  |  |
| Never | 287 (73.0) | 359 (73.0) | 113 (62.8) | 43 (69.4) |
| 1–3 days | 33 (8.4) | 41 (8.3) | 28 (15.6) | 5 (8.1) |
| 1–3 days per week | 22 (5.6) | 23 (4.7) | 9 (5.0) | 3 (4.8) |
| Every or almost every workday | 51 (13.0) | 69 (14.0) | 30 (16.7) | 11 (17.7) |
| Odors from air conditioner |  |  |  |  |
| Never | 315 (80.2) | 395 (80.3) | 135 (75.0) | 45 (72.6) |
| 1–3 days | 41 (10.4) | 53 (10.8) | 17 (9.47) | 5 (8.1) |
| 1–3 days per week | 19 (4.8) | 19 (3.9) | 12 (6.7) | 5 (8.1) |
| Every or almost every workday | 18 (4.6) | 25 (5.1) | 16 (8.9) | 7 (11.3) |
| Mold odor |  |  |  |  |
| Never | 330 (84.0) | 410 (83.3) | 136 (75.6) | 48 (77.4) |
| 1–3 days | 34 (8.7) | 47 (9.6) | 19 (10.6) | 5 (8.1) |
| 1–3 days per week | 15 (3.8) | 15 (3.0) | 9 (5.0) | 4 (6.5) |
| Every or almost every workday | 14 (3.6) | 20 (4.1) | 16 (8.9) | 5 (8.1) |
| Dust and dirt |  |  |  |  |
| Never | 274 (69.7) | 342 (69.5) | 101 (56.1) | 38 (61.3) |
| 1–3 days | 56 (14.2) | 69 (14.0) | 30 (16.7) | 6 (9.7) |
| 1–3 days per week | 31 (7.9) | 34 (6.9) | 16 (8.9) | 6 (9.7) |
| Every or almost every workday | 32 (8.1) | 47 (9.6) | 33 (18.3) | 12 (19.4) |
| Tobacco smoke odor |  |  |  |  |
| Never | 294 (74.8) | 362 (73.6) | 126 (70.0) | 37 (59.7) |
| 1–3 days | 38 (9.7) | 51 (10.4) | 14 (7.8) | 7 (11.3) |
| 1–3 days per week | 25 (6.4) | 34 (6.9) | 12 (6.7) | 10 (16.1) |
| Every or almost every workday | 36 (9.2) | 45 (9.1) | 28 (15.6) | 8 (12.9) |
| Unpleasant chemical odor |  |  |  |  |
| Never | 369 (93.9) | 459 (93.3) | 159 (88.3) | 50 (80.6) |
| 1–3 days | 16 (4.1) | 19 (3.9) | 8 (4.4) | 4 (6.5) |
| 1–3 days per week | 4 (1.0) | 7 (1.4) | 7 (3.9) | 3 (4.8) |
| Every or almost every workday | 4 (1.0) | 7 (1.4) | 6 (3.3) | 5 (8.1) |
| Unpleasant other odor*^f^* |  |  |  |  |
| Never | 279 (71.0) | 331 (67.3) | 113 (62.8) | 30 (48.4) |
| 1–3 days | 47 (12.0) | 64 (13.0) | 20 (11.1) | 10 (16.1) |
| 1–3 days per week | 33 (8.4) | 51 (10.4) | 18 (10.0) | 10 (16.1) |
| Every or almost every workday | 34 (8.7) | 46 (9.3) | 29 (16.1) | 12 (19.4) |
| Job stressors |  |  |  |  |
| Amount of work |  |  |  |  |
| Less/low | 51 (13.0) | 50 (10.2) | 21 (11.8) | 9 (14.3) |
| Somewhat less/low | 71 (18.1) | 92 (18.8) | 30 (16.9) | 13 (20.6) |
| Medium | 149 (37.9) | 176 (36.0) | 71 (39.9) | 24 (38.1) |
| Somewhat more/high | 66 (16.8) | 92 (18.8) | 34 (19.1) | 9 (14.3) |
| More/high | 56 (14.2) | 79 (16.2) | 22 (12.4) | 8 (12.7) |
| Mental workload |  |  |  |  |
| Less/low | 40 (10.2) | 45 (9.2) | 19 (10.6) | 50 (7.9) |
| Somewhat less/low | 119 (30.3) | 142 (29.0) | 62 (34.6) | 24 (38.1) |
| Medium | 146 (37.2) | 180 (36.7) | 57 (31.8) | 21 (33.3) |
| Somewhat more/high | 71 (18.1) | 101 (20.6) | 36 (20.1) | 11 (17.5) |
| More/high | 17 (4.3) | 22 (4.5) | 5 (2.8) | 2 (3.2) |
| Physical overload |  |  |  |  |
| Somewhat less/low | 212 (53.8) | 210 (42.9) | 89 (49.7) | 31 (49.2) |
| Medium | 119 (30.2) | 159 (32.4) | 48 (26.8) | 16 (25.4) |
| Somewhat more/high | 40 (10.2) | 78 (15.9) | 23 (12.8) | 7 (11.1) |
| More/high | 23 (5.8) | 43 (8.8) | 19 (10.6) | 9 (14.3) |
| Interpersonal conflict |  |  |  |  |
| Less/low | 14 (3.6) | 10 (2.0) | 9 (5.1) | 4 (6.5) |
| Somewhat less/low | 82 (20.9) | 64 (13.1) | 26 (14.6) | 6 (9.7) |
| Medium | 172 (43.8) | 203 (41.5) | 78 (43.8) | 23 (37.1) |
| Somewhat more/high | 88 (22.4) | 144 (29.4) | 43 (24.2) | 21 (33.9) |
| More/high | 37 (9.4) | 68 (13.9) | 22 (12.4) | 8 (12.9) |
| Job control |  |  |  |  |
| Less/low | 23 (5.8) | 32 (6.5) | 11 (6.1) | 4 (6.5) |
| Somewhat less/low | 68 (17.3) | 117 (23.9) | 38 (21.2) | 9 (14.5) |
| Medium | 151 (38.3) | 193 (39.5) | 62 (34.6) | 26 (41.9) |
| Somewhat more/high | 114 (28.9) | 113 (23.1) | 54 (30.2) | 17 (27.4) |
| More/high | 38 (9.6) | 34 (7.0) | 14 (7.8) | 6 (9.7) |
| Skill utilization |  |  |  |  |
| Less/low | 40 (10.2) | 50 (10.2) | 24 (13.3) | 6 (9.5) |
| Somewhat less/low | 97 (24.6) | 125 (25.5) | 51 (28.3) | 18 (28.6) |
| Medium | 206 (52.3) | 252 (51.4) | 83 (46.1) | 30 (47.6) |
| Somewhat more/high | 51 (12.9) | 63 (12.9) | 22 (12.2) | 9 (14.3) |
| Job suitability |  |  |  |  |
| Less/low | 42 (10.7) | 50 (10.2) | 22 (12.4) | 6 (9.5) |
| Somewhat less/low | 104 (26.5) | 148 (30.3) | 45 (25.3) | 12 (19.0) |
| Medium | 187 (47.7) | 236 (48.3) | 86 (48.3) | 34 (54.0) |
| More/high | 59 (15.1) | 55 (11.2) | 25 (14.0) | 11 (17.5) |
| Work satisfaction |  |  |  |  |
| Less/low | 53 (13.6) | 78 (16.0) | 24 (13.6) | 6 (9.5) |
| Somewhat less/low | 104 (26.6) | 168 (34.5) | 58 (32.8) | 24 (38.1) |
| Medium | 180 (46.0) | 197 (40.5) | 77 (43.5) | 24 (38.1) |
| More/high | 54 (13.8) | 44 (9.0) | 18 (10.2) | 9 (14.3) |

Values are expressed as number of cases (%) for participants with complete data. Text in parentheses reflects case groups. *^a^* Number of people working in the room in which workstation of respondent is located. *^b^* with cleanser, glue, correction fluid, or other odorous chemicals. *^c^* Change taken place within five meters of workstation in last three months. *^d^* Within two meters of workstation. *^e^* In workplace indoors. *^f^* For example, body odor, food odor, or perfume.
